# RADI: a low-rank ADI-type algorithm for large scale algebraic Riccati equations

**DOI:** 10.1007/s00211-017-0907-5

**Published:** 2017-07-24

**Authors:** Peter Benner, Zvonimir Bujanović, Patrick Kürschner, Jens Saak

**Affiliations:** 1 Max Planck Institute for Dynamics of Complex Technical Systems, Magdeburg, Germany; 2 Department of Mathematics, Faculty of Science, University of Zagreb, Zagreb, Croatia

**Keywords:** 15A24, 15A18, 65F15, 65F30

## Abstract

This paper introduces a new algorithm for solving large-scale continuous-time algebraic Riccati equations (CARE). The advantage of the new algorithm is in its immediate and efficient low-rank formulation, which is a generalization of the Cholesky-factored variant of the Lyapunov ADI method. We discuss important implementation aspects of the algorithm, such as reducing the use of complex arithmetic and shift selection strategies. We show that there is a very tight relation between the new algorithm and three other algorithms for CARE previously known in the literature—all of these seemingly different methods in fact produce exactly the same iterates when used with the same parameters: they are algorithmically different descriptions of the same approximation sequence to the Riccati solution.

## Introduction

The continuous-time algebraic Riccati equation,

1 $$\begin{aligned} A^*X + X A + Q - X G X = 0, \end{aligned}$$

where

$$\begin{aligned} Q = C^*C, \; G = B B^*, \quad A\in {\mathbb {R}}^{n \times n},\; B\in {\mathbb {R}}^{n \times m},\; C\in {\mathbb {R}}^{p \times n}, \end{aligned}$$

appears frequently in various aspects of control theory, such as linear-quadratic optimal regulator problems, $H_2$ and $H_\infty$ controller design and balancing-related model reduction. While the equation may have many solutions, for such applications one is interested in finding a so-called stabilizing solution: the unique positive semidefinite solution $X\in {\mathbb {C}}^{n\times n}$ such that the matrix $A-GX$ is stable (i.e. all of its eigenvalues belong to $\mathbb {C}_-$, the left half of the complex plane). If the pair (*A*, *G*) is stabilizable (i.e. ${\text {rank}}[A-\lambda I, \;G] = n$, for all $\lambda$ in the closed right half plane), and the pair (*A*, *Q*) is detectable (i.e. $(A^*, Q^*)$ is stabilizable), then such a solution exists [11, 19]. These conditions are fulfilled generically, and we assume they hold throughout the paper.

There are several algorithms for finding the numerical solution of (1). In the case when *n* is small enough, one can compute the eigen- or Schur decomposition of the associated Hamiltonian matrix

2 $$\begin{aligned} \mathscr {H} = \left[ \begin{array}{cc} A &{} G \\ Q &{} -A^*\end{array}\right] , \end{aligned}$$

and use an explicit formula for *X*, see [11, 20]. However, if the dimensions of the involved matrices prohibit the computation of a full eigenvalue or Schur decomposition, specialized large-scale algorithms have to be constructed. In such scenarios, Riccati equations arising in applications have additional properties: *A* is sparse, and $p, m \ll n$, thus making the matrices *Q* and *G* of very-low rank compared to *n*. In practice, this often implies that the sought-after solution *X* will have a low numerical rank [3], and allows for construction of iterative methods that approximate *X* with a series of matrices stored in low-rank factored form. Most of these methods are engineered as generalized versions of algorithms for solving a large-scale Lyapunov equation [10, 33], which is a special case of (1) with $G=0$.

The alternating directions implicit (ADI) method [35] is a well established iterative approach for computing solutions of Lyapunov and other linear matrix equations. There exists an array of ADI methods [7, 8, 21, 22, 27, 30, 35, 36], covering both the ordinary and the generalized case. All of these methods have simple statements and efficient implementations [27]. One particular advantage of ADI methods is that they are very well suited for large-scale problems: the default formulation which works with full-size dense matrices can be transformed into a series of iteratively built approximations to the solution. Such approximations are represented in factored form, each factor having a very small rank compared to the dimensions of the input matrices. This makes ADI methods very suitable for large-scale applications.

Recently, Wong and Balakrishnan [37, 38] suggested a so-called quadratic ADI method (qADI) for solving the algebraic Riccati equation (1). Their method is a direct generalization of the Lyapunov ADI method, but only when considering the formulation working with full-size dense matrices. However, in the setting of the qADI algorithm, it appears impossible to apply a so-called “Li–White trick” [22], which is the usual method of obtaining a low-rank formulation of an ADI method. Wong and Balakrishnan do provide a low-rank variant of their algorithm, but this variant has an important drawback: in each step, all the low-rank factors have to be rebuilt from scratch. This has a large negative impact on the performance of the algorithm.

Apart from the qADI method, there are several other methods for solving the large-scale Riccati equation that have appeared in the literature recently. Amodei and Buchot [1] derive an approximation of the solution by computing small-dimensional invariant subspaces of the associated Hamiltonian matrix (2). Lin and Simoncini [23] also consider the Hamiltonian matrix, and construct the solution by running subspace iterations on its Cayley transforms. Massoudi et al. [24] have shown that the latter method can be obtained from the control theory point of view as well.

In this paper, we introduce a new ADI-type iteration for Riccati equations, RADI. The derivation of RADI is not related to qADI, and it immediately gives the low-rank algorithm which overcomes the drawback from [37, 38]. The low-rank factors are built incrementally in the new algorithm: in each step, each factor is expanded by several columns and/or rows, while keeping the elements from the previous steps intact. By setting the quadratic coefficient *B* in (1) to zero, our method reduces to the low-rank formulation of the Lyapunov ADI method, see, e.g., [4, 7, 22, 27].

A surprising result is that, despite their completely different derivations, all of the Riccati methods we mentioned so far are equivalent: the approximations they produce in each step are the same. This was already shown [3] for the qADI algorithm, and the algorithm of Amodei and Buchot. In this paper we extend this equivalence to our new low-rank RADI method and the method of Lin and Simoncini. Among all these different formulations of the same approximation sequence, RADI offers a compact and efficient implementation, and is very well suited for effective computation.

This paper is organized as follows: in Sect. 2, we recall the statements of the Lyapunov ADI method and the various Riccati methods, and introduce the new low-rank RADI algorithm. The equivalence of all aforementioned methods is shown in Sect. 3. In Sect. 4 we discuss important implementation issues, and in particular, various strategies for choosing shift parameters. Finally, Sect. 5 compares the effect of different options for the algorithm on its performance via several numerical experiments. We compare RADI with other algorithms for computing low-rank approximate solutions of (1) as well: the extended [16] and rational Krylov subspace methods [34], and the low-rank Newton–Kleinman ADI iteration [7, 9, 15, 29].

The following notation is used in this paper: $\mathbb {C}_-$ and $\mathbb {C}_+$ are the open left and right half plane, respectively, while ${\text {Re}}\left( z\right) ,~{\text {Im}}\left( z\right)$, $\overline{z}={\text {Re}}\left( z\right) -\mathsf {i}{\text {Im}}\left( z\right)$, |*z*| denote the real part, imaginary part, complex conjugate, and absolute value of a complex quantity *z*. For the matrix *A*, we use $A^*$ and $A^{-1}$ for the complex conjugate transpose and the inverse, respectively. In most situations, expressions of the form $x=A^{-1}b$ are to be understood as solving the linear system $Ax=b$ of equations for *b*. The relations $A>(\ge )0$, $A<(\le )0$ stand for the matrix *A* being positive or negative (semi)definite. Likewise, $A\ge (\le ) B$ refers to $A-B\ge (\le )0$. If not stated otherwise, $\Vert \cdot \Vert$ is the Euclidean vector or subordinate matrix norm, and $\kappa (\cdot )$ is the associated condition number.

## A new low-rank factored iteration

We start this section by stating various methods for solving Lyapunov and Riccati equations, which will be used throughout the paper. First, consider the Lyapunov equation

3 $$\begin{aligned} A^*X^{\mathsf {lya}}+ X^{\mathsf {lya}}A + Q = 0, \quad Q = C^*C, \quad A\in {\mathbb {R}}^{n \times n},\; C\in {\mathbb {R}}^{p \times n}. \end{aligned}$$

Here we assume that *n* is much larger than *p*. When *A* is a stable matrix, the solution $X^{\mathsf {lya}}$ is positive semidefinite. The Lyapunov ADI algorithm [36] generates a sequence of approximations $(X^{\mathsf {lya}}_{k})_k$ to $X^{\mathsf {lya}}$ defined by

4 $$\begin{aligned} \left. \begin{array}{rcl} X^{\mathsf {lya}}_{k+1/2} (A+\overline{\sigma _{k+1}} I) &{}=&{} -Q - (A^*- \overline{\sigma _{k+1}} I)X^{\mathsf {lya}}_{k}, \\ (A^*+ \sigma _{k+1} I) X^{\mathsf {lya}}_{k+1} &{}=&{} -Q - X^{\mathsf {lya}}_{k+1/2} (A - \sigma _{k+1} I). \end{array} \right\} \text { Lyapunov ADI} \end{aligned}$$

We will assume that the initial iterate $X^{\mathsf {lya}}_{0}$ is the zero matrix, although it may be set arbitrarily. The complex numbers $\sigma _k \in \mathbb {C}_-$ are called shifts, and the performance of ADI algorithms depends strongly on the appropriate selection of these parameters [30]; this is further discussed in the context of the RADI method in Sect. 4.5. Since each iteration matrix $X^{\mathsf {lya}}_{k}$ is of order *n*, formulation (4) is unsuitable for large values of *n*, due to the amount of memory needed for storing $X^{\mathsf {lya}}_{k}\in {\mathbb {C}}^{n \times n}$ and to the computational time needed for solving *n* linear systems in each half-step. The equivalent low-rank algorithm [4, 6] generates the same sequence, but represents $X^{\mathsf {lya}}_{k}$ in factored form $X^{\mathsf {lya}}_{k}= Z^{\mathsf {lya}}_{k}(Z^{\mathsf {lya}}_{k})^*$ with $Z^{\mathsf {lya}}_{k}\in {\mathbb {C}}^{n \times pk}$:

5 $$\begin{aligned} \left. \begin{array}{rcl} R^{\mathsf {lya}}_{0} &{}=&{} C^*, \\ V^{\mathsf {lya}}_{k} &{}=&{} \sqrt{-2{\text {Re}}\left( \sigma _k\right) } \cdot (A^*+ \sigma _k I)^{-1}R^{\mathsf {lya}}_{k-1}, \\ R^{\mathsf {lya}}_{k} &{}=&{} R^{\mathsf {lya}}_{k-1} + \sqrt{-2{\text {Re}}\left( \sigma _k\right) } \cdot V^{\mathsf {lya}}_{k}, \\ Z^{\mathsf {lya}}_{k} &{}=&{} \left[ \begin{array}{cc} Z^{\mathsf {lya}}_{k-1} &{} V^{\mathsf {lya}}_{k} \end{array}\right] . \end{array} \right\} \text { low-rank Lyapunov ADI} \end{aligned}$$

Initially, the matrix $Z^{\mathsf {lya}}_{0}$ is empty. This formulation is far more efficient for large values of *n*, since the right-hand side of the linear system in each step involves only the tall-and-skinny matrix $R^{\mathsf {lya}}_{k-1} \in {\mathbb {C}}^{n \times p}$.

We now turn our attention to the Riccati equation (1). Once again we assume that $p, m \ll n$, and seek to approximate the stabilizing solution *X*. Wong and Balakrishnan [37, 38] suggest the following quadratic ADI iteration (abbreviated as qADI):

6 $$\begin{aligned} \left. \begin{array}{rcl} X^{\mathsf {adi}}_{k+1/2} \left( A+\overline{\sigma _{k+1}} I - G X^{\mathsf {adi}}_{k}\right) &{}=&{} -Q - (A^*- \overline{\sigma _{k+1}} I)X^{\mathsf {adi}}_{k}, \\ \left( A^*+ \sigma _{k+1} I - X^{\mathsf {adi}}_{k+1/2} G\right) X^{\mathsf {adi}}_{k+1} &{}=&{} -Q - X^{\mathsf {adi}}_{k+1/2} \left( A - \sigma _{k+1} I\right) . \end{array} \right\} \text { quadratic ADI} \end{aligned}$$

Again, the initial approximation is usually set to $X^{\mathsf {adi}}_{0}=0$. Note that by inserting $G=0$ in the quadratic iteration we obtain the Lyapunov ADI algorithm (4). As mentioned in the introduction, we will develop a low-rank variant of this algorithm such that inserting $G=0$ will reduce it precisely to (5). To prepare the terrain, we need to introduce two more methods for solving the Riccati equation.

In the small scale setting, the Riccati equation (1) is usually solved by computing the stable invariant subspace of the associated $2n \times 2n$ Hamiltonian matrix (2). To be more precise, let $1 \le k \le n$, and

7 $$\begin{aligned} \mathscr {H} \left[ \begin{array}{c} P_k \\ Q_k \end{array}\right] = \left[ \begin{array}{cc} A &{} G \\ Q &{} -A^*\end{array}\right] \left[ \begin{array}{c} P_k \\ Q_k \end{array}\right] = \left[ \begin{array}{c} P_k \\ Q_k \end{array}\right] \varLambda _k, \end{aligned}$$

where $P_k, Q_k \in {\mathbb {C}}^{n \times k}$ and the matrix $\varLambda _k \in {\mathbb {C}}^{k \times k}$ is stable. For $k=n$, the stabilizing solution of (1) is given by $X = -Q_k P_k^{-1}$. In the large scale setting, it is computationally too expensive to compute the entire *n*-dimensional stable invariant subspace of $\mathscr {H}$. Thus an alternative approach was suggested in [1]: for $k \ll n$, one can compute only a *k*-dimensional, stable, invariant subspace and use an approximation given by the formula

8 $$\begin{aligned} \left. X^{\mathsf {inv}}_{k}= -Q_k (Q_k^*P_k)^{-1} Q_k^*. \quad \right\} \text { invariant subspace approach} \end{aligned}$$

Clearly, $X^{\mathsf {inv}}_{n} = X$. This approach was further studied and justified in [3], where it was shown that

$$\begin{aligned} X^{\mathsf {inv}}_{k}= X^{\mathsf {adi}}_{k}, \text { for all } k, \end{aligned}$$

if $p=1$ and the shifts used for the qADI iteration coincide with the Hamiltonian eigenvalues of the matrix $\varLambda _k$. In fact, properties of the approximate solution $X^{\mathsf {inv}}_{k}$ given in [3] have lead us to the definition of the low-rank variant of the qADI iteration that is described in this paper.

The final method we consider also uses the Hamiltonian matrix $\mathscr {H}$. The Cayley transformed Hamiltonian subspace iteration introduced in [23] generates a sequence of approximations $(X^{\mathsf {cay}}_{k})_k$ for the stabilizing solution of the Riccati equation defined by

9 $$\begin{aligned} \left. \begin{array}{rcl} \left[ \begin{array}{c} M_k \\ N_k \end{array}\right] &{}=&{} (\mathscr {H} - \sigma _k I)^{-1} (\mathscr {H} + \overline{\sigma _k}I) \left[ \begin{array}{c} I \\ -X^{\mathsf {cay}}_{k-1} \end{array}\right] ,\\ X^{\mathsf {cay}}_{k}&{}=&{} -N_k M_k^{-1}, \end{array} \right\} \text { Cayley subspace iteration} \end{aligned}$$

Here $X^{\mathsf {cay}}_{0}$ is some initial approximation and $\sigma _k \in \mathbb {C}_-$ are any chosen shifts (Here we have adapted the notation to fit the one of this paper). In Sect. 3 we will show that this method is also equivalent to the qADI and the new low-rank RADI iterations.

### Derivation of the algorithm

The common way of converting ADI iterations into their low-rank variants is to perform a procedure similar to the one originally done by Li and White [22] for the Lyapunov ADI method. A crucial assumption for this procedure to succeed is that the matrices participating in the linear systems in each of the half-steps mutually commute for all *k*. For the Lyapunov ADI method this obviously holds true for the matrices $A^*+ \sigma _{k+1} I$. However, in the case of the quadratic ADI iteration (6), the matrices $A^*+ \sigma _{k+1} I - X^{\mathsf {adi}}_{k} G$ do not commute in general, for all *k*, and neither do $A^*+ \sigma _{k+1} I - X^{\mathsf {adi}}_{k+1/2} G$.

Thus we take a different approach in constructing the low-rank version. A common way to measure the quality of the matrix $\varXi \in {\mathbb {C}}^{n \times n}$ as an approximation to the Riccati solution is to compute the norm of its residual matrix

$$\begin{aligned} \mathscr {R}(\varXi ) = A^*\varXi + \varXi A + Q - \varXi G \varXi . \end{aligned}$$

The idea for our method is to repetitively update the approximation $\varXi$ by forming a so-called residual equation, until its solution converges to zero. The background is given in the following simple result.

#### Theorem 1

Let $\varXi \in {\mathbb {C}}^{n\times n}$ be an approximation to a solution of (1).

- Let $X=\varXi + {\tilde{X}}$ be an exact solution of (1). Then ${\tilde{X}}$ is a solution to the residual equation
  10 $$\begin{aligned} \tilde{A}^*{\tilde{X}} + {\tilde{X}} \tilde{A} + \tilde{Q} - {\tilde{X}}G{\tilde{X}} = 0, \end{aligned}$$
  where $$\tilde{A} = A - G \varXi $$ and $$\tilde{Q} = \mathscr {R}(\varXi )$$.
- Conversely, if ${\tilde{X}}$ is a solution to (10), then $X = \varXi + {\tilde{X}}$ is a solution to the original Riccati equation (1). Moreover, if $\varXi \ge 0$ and ${\tilde{X}}$ is a stabilizing solution to (10), then $X = \varXi + {\tilde{X}}$ is the stabilizing solution to (1).
- If $\varXi \ge 0$ and $\mathscr {R}(\varXi ) \ge 0$, then the residual equation (10) has a unique stabilizing solution.
- If $\varXi \ge 0$ and $\mathscr {R}(\varXi ) \ge 0$, then $\varXi \le X$, where *X* is the stabilizing solution of (1).

#### Proof

- This is a straightforward computation which follows by inserting ${\tilde{X}} = X-\varXi$ and the formula for the residual of $\varXi$ into (10), see also [2, 25].
- The first part follows as in (a). If $\varXi \ge 0$ and ${\tilde{X}}$ is a stabilizing solution to (10), then $X = \varXi + {\tilde{X}} \ge 0$ and $A-GX = \tilde{A} - G{\tilde{X}}$ is stable, which makes *X* the stabilizing solution to (1).
- The claims follow directly from (a) and [19, Theorem 9.1.1].

$$\square $$

Our algorithm will have the following form:

1. Let $$\varXi =0$$.
2. Form the residual equation (10) for the approximation $\varXi$.
3. Compute an approximation ${\tilde{X}}_1 \approx {\tilde{X}}$, where ${\tilde{X}}$ is the stabilizing solution of (10).
4. Accumulate $\varXi \leftarrow \varXi + {\tilde{X}}_1$, and go to Step 2.

To complete the derivation, we need to specify Step 3 in a way that ${\tilde{X}}_1 \ge 0$ and $\mathscr {R}(\varXi + {\tilde{X}}_1) \ge 0$. With these two conditions imposed, Theorem 1 ensures that the residual equation in Step 2 always has a unique stabilizing solution and that the approximation $\varXi$ is kept positive semidefinite and monotonically increasing towards the stabilizing solution of (1). The matrix ${\tilde{X}}_1$ fulfilling these conditions can be obtained by computing a 1-dimensional invariant subspace for the Hamiltonian matrix associated with the residual equation, and plugging it into formula (8).

More precisely, assume that $\mathscr {R}(\varXi ) = {\tilde{C}}^*{\tilde{C}} \ge 0$, $G = BB^*$, and that $r, q \in {\mathbb {C}}^n$ satisfy

$$\begin{aligned} \left[ \begin{array}{cc} \tilde{A} &{} BB^*\\ {\tilde{C}}^*{\tilde{C}} &{} -\tilde{A}^*\end{array}\right] \left[ \begin{array}{c} r \\ q \end{array}\right] = \lambda \left[ \begin{array}{c} r \\ q \end{array}\right] , \end{aligned}$$

where $\lambda \in \mathbb {C}_-$ is such that $-\lambda$ is not an eigenvalue of $\tilde{A}$. Equivalently,

$$\begin{aligned} {\tilde{A}} r + BB^*q&= \lambda r, \\ {\tilde{C}}^*{\tilde{C}} r - {\tilde{A}}^*q&= \lambda q. \end{aligned}$$

From the second equation we get $q = ({\tilde{A}}^*+ \lambda I)^{-1} {\tilde{C}}^*({\tilde{C}}r)$. Let

$$\begin{aligned} {\tilde{V}}_1 = \sqrt{-2{\text {Re}}\left( \lambda \right) }({\tilde{A}}^*+ \lambda I)^{-1} {\tilde{C}}^*. \end{aligned}$$

Multiply the first equation by $q^*$ from the left, and the transpose of the second by *r* from the right; then add the terms to obtain

$$\begin{aligned} q^*r= & {} \frac{1}{2{\text {Re}}\left( \lambda \right) } (q^*BB^*q + r^*{\tilde{C}}^*{\tilde{C}} r) \nonumber \\= & {} \frac{1}{2{\text {Re}}\left( \lambda \right) } ({\tilde{C}}r)^*\left( I - \frac{1}{2{\text {Re}}\left( \lambda \right) } \left( {\tilde{V}}_1^*B\right) \left( {\tilde{V}}_1^*B\right) ^*\right) ({\tilde{C}}r). \end{aligned}$$

Expression (8) has the form ${\tilde{X}}_1 = -q (q^*r)^{-1}q^*$. When $p=1$ and ${\tilde{C}}r \ne 0$, the terms containing ${\tilde{C}}r$ cancel out, and we get

11 $$\begin{aligned} \left. \begin{array}{rcl} {\tilde{V}}_1 &{}=&{} \sqrt{-2{\text {Re}}\left( \lambda \right) } \cdot ({\tilde{A}}^*+ \lambda I)^{-1} {\tilde{C}}^*, \\ {\tilde{Y}}_1 &{}=&{} I - \frac{1}{2{\text {Re}}\left( \lambda \right) } \left( {\tilde{V}}_1^*B\right) \left( {\tilde{V}}_1^*B\right) ^*, \\ {\tilde{X}}_1 &{}=&{} {\tilde{V}}_1 {\tilde{Y}}_1^{-1} {\tilde{V}}_1^*. \end{array} \right\} \end{aligned}$$

The derivation above is valid when $\lambda$ is an eigenvalue of the Hamiltonian matrix, similarly as in [3, Theorem 7] which also studies residual Riccati equations related to invariant subspaces of Hamiltonian matrices. Nevertheless, the expression (11) is well-defined even when $\lambda$ is not an eigenvalue of the Hamiltonian matrix, and for $p>1$ as well. The Hamiltonian argument here serves only as a motivation for introducing (11), and the following proposition shows that the desired properties of the updated matrix $\varXi + {\tilde{X}}_1$ still hold for any $\lambda$ in the left half-plane which is not an eigenvalue of $-{\tilde{A}}$, and for all *p*.

#### Proposition 1

Let $\varXi \ge 0$ be such that $\mathscr {R}(\varXi )={\tilde{C}}^*{\tilde{C}} \ge 0$, and let $\lambda \in \mathbb {C}_-$ not be an eigenvalue of $-{\tilde{A}}$. The following holds true for the update matrix ${\tilde{X}}_1$ as defined in (11):

- $${\tilde{X}}_1 \ge 0$$, i.e. $$\varXi + {\tilde{X}}_1 \ge 0$$.
- $$\mathscr {R}(\varXi + {\tilde{X}}_1) = \hat{C}^*\hat{C} \ge 0$$, where $$\hat{C}^*= {\tilde{C}}^*+ \sqrt{-2{\text {Re}}\left( \lambda \right) } \cdot {\tilde{V}}_1 {\tilde{Y}}_1^{-1}$$.

#### Proof

- Positive definiteness of ${\tilde{Y}}_1$ (and then the semi-definiteness of ${\tilde{X}}_1$ as well) follows directly from ${\text {Re}}\left( \lambda \right) < 0$.
- Note that ${\tilde{A}}^*{\tilde{V}}_1 = \sqrt{-2{\text {Re}}\left( \lambda \right) } \cdot {\tilde{C}}^*- \lambda {\tilde{V}}_1$, and $({\tilde{V}}_1^*B)({\tilde{V}}_1^*B)^*= 2{\text {Re}}\left( \lambda \right) I - 2{\text {Re}}\left( \lambda \right) {\tilde{Y}}_1$. We use these expressions to obtain:
  $$\begin{aligned} \mathscr {R}(\varXi + {\tilde{X}}_1)&={\tilde{A}}^*{\tilde{X}}_1 + {\tilde{X}}_1 {\tilde{A}} + \mathscr {R}(\varXi ) - {\tilde{X}}_1 BB^*{\tilde{X}}_1 \\&= \left( {\tilde{A}}^*{\tilde{V}}_1\right) {\tilde{Y}}_1^{-1} {\tilde{V}}_1^*+ {\tilde{V}}_1 {\tilde{Y}}_1^{-1} \left( {\tilde{A}}^*{\tilde{V}}_1\right) ^*+ {\tilde{C}}^*{\tilde{C}} \\&\quad \,- {\tilde{V}}_1 {\tilde{Y}}_1^{-1} \left( {\tilde{V}}_1^*B\right) \left( {\tilde{V}}_1^*B\right) ^*{\tilde{Y}}_1^{-1} {\tilde{V}}_1^*\\&= \sqrt{-2{\text {Re}}\left( \lambda \right) } \cdot {\tilde{C}}^*{\tilde{Y}}_1^{-1} {\tilde{V}}_1^*+ \sqrt{-2{\text {Re}}\left( \lambda \right) } \cdot {\tilde{V}}_1 {\tilde{Y}}_1^{-1} {\tilde{C}} + {\tilde{C}}^*{\tilde{C}} \\&\quad \, - 2{\text {Re}}\left( \lambda \right) {\tilde{V}}_1 {\tilde{Y}}_1^{-1} {\tilde{Y}}_1^{-1} {\tilde{V}}_1^*\\&= \left( {\tilde{C}}^*+ \sqrt{-2{\text {Re}}\left( \lambda \right) }\cdot {\tilde{V}}_1 {\tilde{Y}}_1^{-1}\right) \cdot \left( {\tilde{C}}^*+ \sqrt{-2{\text {Re}}\left( \lambda \right) }\cdot {\tilde{V}}_1 {\tilde{Y}}_1^{-1}\right) ^*. \end{aligned}$$

$$\square $$

We are now ready to state the new RADI algorithm. Starting with the initial approximation $X_0=0$ and the residual $\mathscr {R}(X_0)=C^*C$, we continue by selecting a shift $\sigma _k\in \mathbb {C}_-$ and computing the approximation $X_k = Z_k Y_k^{-1} Z_k^*$ with the residual $\mathscr {R}(X_k) = R_k R_k^*$, for $k=1, 2, \ldots$. The transition from $X_{k-1}$ to $X_k = X_{k-1} + V_k {\tilde{Y}}_k^{-1} V_k^*$ is computed via (11), adapted to approximate the solution of the residual equation with $\varXi = X_{k-1}$, i.e.  with ${\tilde{A}} = A - BB^*X_{k-1}$ and ${\tilde{C}}^*= R_{k-1}$. Proposition 1 provides a very efficient update formula for the low-rank factor $R_k$ of the residual. The whole procedure reduces to the following:

12 $$\begin{aligned} \left. \begin{array}{rcl} R_0 &{}=&{} C^*, \\ V_k &{}=&{} \sqrt{-2{\text {Re}}\left( \sigma _k\right) } \cdot (A^*- X_{k-1} BB^*+ \sigma _k I)^{-1} R_{k-1}, \\ {\tilde{Y}}_k &{}=&{} I - \frac{1}{2{\text {Re}}\left( \sigma _k\right) } \left( V_k^*B\right) \left( V_k^*B\right) ^*; \quad Y_k = \left[ \begin{array}{cc} Y_{k-1} &{} \\ &{} {\tilde{Y}}_k \end{array}\right] , \\ R_k &{}=&{} R_{k-1} + \sqrt{-2{\text {Re}}\left( \sigma _k\right) } \cdot V_k {\tilde{Y}}_k^{-1}, \\ Z_k &{}=&{} \left[ \begin{array}{cc} Z_{k-1} &{} V_k \end{array}\right] . \end{array} \right\} \text {RADI iteration} \end{aligned}$$

Note that any positive semi-definite $X_0$ can be used as an initial approximation, as long as its residual is positive semi-definite as well, and its low-rank Cholesky factorization can be computed. From the derivation of the RADI algorithm we have that

$$\begin{aligned} X_k = \sum _{i=1}^k V_i {\tilde{Y}}_i^{-1} V_i^*; \end{aligned}$$

in formulation (12) of the method we have collected $V_1, \ldots , V_k$ into the matrix $Z_k$, and ${\tilde{Y}}_1, \ldots , {\tilde{Y}}_k$ into the block-diagonal matrix $Y_k$.

When $p=1$ and all the shifts are chosen as eigenvalues of the Hamiltonian matrix associated with the initial Riccati equation (1), the update described in Proposition 1 reduces to [3, Theorem 5]. Thus in that case, the RADI algorithm reduces to the invariant subspace approach (8).

Furthermore, iteration (12) clearly reduces to the low-rank Lyapunov ADI method (5) when $B=0$; in that case $Y_k = I$. The relation to the original qADI iteration (6) is not clear unless $p=1$ and the shifts are chosen as eigenvalues of $\mathscr {H}$, in which case both of these methods coincide with the invariant subspace approach. We discuss this further in the following section.

## Equivalences with other Riccati methods

In this section we prove that all Riccati solvers introduced in Sect. 2 in fact compute exactly the same iterations, which we will refer to as the Riccati ADI iterations in the remaining text. This result is collected in Theorem 2; we begin with a simple technical lemma that provides different representations of the residual factor.

### Lemma 1

Let

$$\begin{aligned} R_k^{(1)}&= \frac{1}{\sqrt{-2{\text {Re}}\left( \sigma _k\right) }} \cdot (A^*- X_k G - \overline{\sigma _k}I ) V_k, \\ R_k^{(2)}&= \frac{1}{\sqrt{-2{\text {Re}}\left( \sigma _{k+1}\right) }} \cdot (A^*- X_k G + \sigma _{k+1}I ) V_{k+1}. \end{aligned}$$

Then $$R_k^{(1)} = R_k^{(2)} = R_k$$.

### Proof

From the definition of the RADI iteration (12) it is obvious that $R_k^{(2)} = R_k$. Using $X_k = X_{k-1} + V_k {\tilde{Y}}_k^{-1} V_k^*$, and $V_k^*G V_k = 2{\text {Re}}\left( \sigma _k\right) I - 2{\text {Re}}\left( \sigma _k\right) {\tilde{Y}}_k$, we have

$$\begin{aligned} R_k&= R_{k-1} + \sqrt{-2{\text {Re}}\left( \sigma _k\right) } V_k {\tilde{Y}}_k^{-1} \\&= \frac{1}{\sqrt{-2{\text {Re}}\left( \sigma _{k}\right) }} \cdot (A^*- X_{k-1} G + \sigma _{k}I ) V_{k} + \sqrt{-2{\text {Re}}\left( \sigma _k\right) } V_k {\tilde{Y}}_k^{-1} \\&= \frac{1}{\sqrt{-2{\text {Re}}\left( \sigma _{k}\right) }} \cdot (A^*- X_{k} G - \overline{\sigma _{k}}I ) V_{k} + \frac{1}{\sqrt{-2{\text {Re}}\left( \sigma _{k}\right) }} V_k{\tilde{Y}}_k^{-1} V_k^*G V_k \\&\quad \quad - \sqrt{-2{\text {Re}}\left( \sigma _k\right) } V_k + \sqrt{-2{\text {Re}}\left( \sigma _k\right) } V_k {\tilde{Y}}_k^{-1} \\&= R^{(1)}_{k}. \end{aligned}$$

$$\square $$

### Theorem 2

If the initial approximation in all algorithms is zero, and the same shifts are used, then for all *k*,

$$\begin{aligned} X_k = X^{\mathsf {adi}}_{k}= X^{\mathsf {cay}}_{k}. \end{aligned}$$

If ${\text {rank}}C = 1$ and the shifts are equal to distinct eigenvalues of $\mathscr {H}$, then for all *k*,

$$\begin{aligned} X_k = X^{\mathsf {adi}}_{k}= X^{\mathsf {cay}}_{k}= X^{\mathsf {inv}}_{k}. \end{aligned}$$

### Proof

We first use induction to show that $X_k = X^{\mathsf {adi}}_{k}$, for all *k*.

Assume that $X_{k-1} = X^{\mathsf {adi}}_{k-1}$. We need to show that $X_k = X_{k-1} + V_k {\tilde{Y}}_k^{-1} V_k^*$ satisfies the defining equality (6) of the qADI iteration, i.e. that

13 $$\begin{aligned} \left( A^*+ \sigma _{k} I - X^{\mathsf {adi}}_{k-1/2} G\right) \left( X_{k-1} + V_k {\tilde{Y}}_k^{-1} V_k^*\right) = -Q - X^{\mathsf {adi}}_{k-1/2} (A - \sigma _{k} I), \end{aligned}$$

where

14 $$\begin{aligned} X^{\mathsf {adi}}_{k-1/2} (A+\overline{\sigma _{k}} I - G X_{k-1}) = -Q - (A^*- \overline{\sigma _{k}} I) X_{k-1}. \end{aligned}$$

First, note that (14) can be rewritten as

$$\begin{aligned} A^*X_{k-1} + X^{\mathsf {adi}}_{k-1/2} A + Q - X^{\mathsf {adi}}_{k-1/2} G X_{k-1} + \overline{\sigma _k}\left( X^{\mathsf {adi}}_{k-1/2} - X_{k-1}\right) = 0. \end{aligned}$$

Subtracting this from the expression for the Riccati residual,

$$\begin{aligned} A^*X_{k-1} + X_{k-1} A + Q - X_{k-1} G X_{k-1} = \mathscr {R}(X_{k-1}), \end{aligned}$$

we obtain

15 $$\begin{aligned} X^{\mathsf {adi}}_{k-1/2} - X_{k-1} = -\mathscr {R}(X_{k-1}) \cdot \left( A - GX_{k-1} + \overline{\sigma _k}I\right) ^{-1}. \end{aligned}$$

Equation (13) can be reorganized as

$$\begin{aligned}&(A^*+ \sigma _{k} I ) \left( X_{k-1} + V_k {\tilde{Y}}_k^{-1} V_k^*\right) - X^{\mathsf {adi}}_{k-1/2} G V_k {\tilde{Y}}_k^{-1} V_k^*\\&\quad = -Q - X^{\mathsf {adi}}_{k-1/2} (A +\overline{\sigma _{k}} I - G X_{k-1} ) + 2{\text {Re}}\left( \sigma _{k}\right) X^{\mathsf {adi}}_{k-1/2}. \end{aligned}$$

Replace the second term on the right-hand side with the right-hand side of (14). Thus, it remains to prove

$$\begin{aligned}&(A^*+ \sigma _{k} I ) \left( X_{k-1} + V_k {\tilde{Y}}_k^{-1} V_k^*\right) - X^{\mathsf {adi}}_{k-1/2} G V_k {\tilde{Y}}_k^{-1} V_k^*\\&\quad = (A^*- \overline{\sigma _{k}} I) X_{k-1} + 2{\text {Re}}\left( \sigma _{k}\right) X^{\mathsf {adi}}_{k-1/2}, \end{aligned}$$

or after some rearranging, and by using (15),

16 $$\begin{aligned}&(A^*- X_{k-1}G + \sigma _k I)V_k {\tilde{Y}}_k^{-1} V_k^*\nonumber \\&\quad = \left( X^{\mathsf {adi}}_{k-1/2} - X_{k-1}\right) \cdot \left( 2{\text {Re}}\left( \sigma _k\right) I + G V_k {\tilde{Y}}_k^{-1} V_k^*\right) \nonumber \\&\quad = -\mathscr {R}(X_{k-1}) \cdot (A - GX_{k-1} + \overline{\sigma _k}I)^{-1} \cdot \left( 2{\text {Re}}\left( \sigma _k\right) I + G V_k {\tilde{Y}}_k^{-1} V_k^*\right) . \end{aligned}$$

Next we use the expression $\mathscr {R}(X_{k-1}) = R_{k-1}^{(2)}(R_{k-1}^{(2)})^*$ of Lemma 1. The right-hand side of (16) is, thus, equal to

$$\begin{aligned} \frac{1}{2{\text {Re}}\left( \sigma _{k}\right) } \cdot (A^*- X_{k-1} G + \sigma _{k}I ) V_{k} V_k^*\cdot \left( 2{\text {Re}}\left( \sigma _k\right) I + G V_k {\tilde{Y}}_k^{-1} V_k^*\right) , \end{aligned}$$

which turns out to be precisely the same as the left-hand side of (16) once we use the identity

$$\begin{aligned} V_k^*G V_k = 2{\text {Re}}\left( \sigma _k\right) I - 2{\text {Re}}\left( \sigma _k\right) {\tilde{Y}}_k. \end{aligned}$$

This completes the proof of $X_k = X^{\mathsf {adi}}_{k}$.

Next, we use induction once again to show $X^{\mathsf {cay}}_{k}= X^{\mathsf {adi}}_{k}$, for all *k*. For $k=0$, the claim is trivial; assume that $X^{\mathsf {cay}}_{k-1} = X^{\mathsf {adi}}_{k-1}$ for some $k \ge 1$. To show that $X^{\mathsf {cay}}_{k}= X^{\mathsf {adi}}_{k}$, let us first multiply (9) by $\mathscr {H}-\sigma _k I$ from the left:

17 $$\begin{aligned} \left[ \begin{array}{cc} A - \sigma _k I &{} G \\ Q &{} -A^*- \sigma _k I \end{array}\right] \left[ \begin{array}{c} M_k\\ N_k \end{array}\right]&= \left[ \begin{array}{cc} A + \overline{\sigma _k} I &{} G \\ Q &{} -A^*+ \overline{\sigma _k} I \end{array}\right] \left[ \begin{array}{c} I \\ -X^{\mathsf {cay}}_{k-1} \end{array}\right] \nonumber \\&=: \left[ \begin{array}{c} M_{k-1/2}\\ N_{k-1/2} \end{array}\right] , \end{aligned}$$

and suggestively introduce $X^{\mathsf {cay}}_{k-1/2} := -N_{k-1/2} M_{k-1/2}^{-1}$. We thus have

18 $$\begin{aligned} A + \overline{\sigma _k} I - G X^{\mathsf {cay}}_{k-1}= & {} M_{k-1/2}, \end{aligned}$$

19 $$\begin{aligned} Q - (-A^*+ \overline{\sigma _k} I) X^{\mathsf {cay}}_{k-1}= & {} N_{k-1/2}, \end{aligned}$$

and

$$\begin{aligned} X^{\mathsf {cay}}_{k-1/2} \left( A + \overline{\sigma _k} I - G X^{\mathsf {cay}}_{k-1}\right) = -N_{k-1/2} M_{k-1/2}^{-1} M_{k-1/2} = -Q - (A^*- \overline{\sigma _k} I) X^{\mathsf {cay}}_{k-1}. \end{aligned}$$

This is the same relation as the one defining $X^{\mathsf {adi}}_{k-1/2}$, and thus $X^{\mathsf {cay}}_{k-1/2} = X^{\mathsf {adi}}_{k-1/2}$. Next, equating the leftmost and the rightmost matrix in (17), it follows that

20 $$\begin{aligned} (A - \sigma _k I) M_k + GN_k= & {} M_{k-1/2},\end{aligned}$$

21 $$\begin{aligned} Q M_k + (-A^*- \sigma _k I) N_k= & {} N_{k-1/2}. \end{aligned}$$

Multiply (21) from the right by $M_k^{-1}$ to obtain

22 $$\begin{aligned} (A^*+ \sigma _k I) X^{\mathsf {cay}}_{k}= -Q + N_{k-1/2}M_k^{-1}, \end{aligned}$$

and multiply (20) from the left by $X^{\mathsf {cay}}_{k-1/2}$ and from the right by $M_k^{-1}$ to get

23 $$\begin{aligned} -X^{\mathsf {cay}}_{k-1/2}GX^{\mathsf {cay}}_{k}= -X^{\mathsf {cay}}_{k-1/2} (A-\sigma _k I) - N_{k-1/2}M_k^{-1}. \end{aligned}$$

Adding (22) and (23) yields

$$\begin{aligned} (A^*+ \sigma _k I - X^{\mathsf {cay}}_{k-1/2}G) X^{\mathsf {cay}}_{k}= -Q - X^{\mathsf {cay}}_{k-1/2} (A-\sigma _k I), \end{aligned}$$

which is the same as the defining equation for $X^{\mathsf {adi}}_{k}$. Thus $X^{\mathsf {cay}}_{k}= X^{\mathsf {adi}}_{k}$, so both the Cayley subspace iteration and the qADI iteration generate the same sequences.

In the case of ${\text {rank}}{C}=1$ and shifts equal to the eigenvalues of $\mathscr {H}$, the equality $X^{\mathsf {inv}}_{k}= X^{\mathsf {adi}}_{k}$ is already shown in [3]. Equality among the iterates generated by the other methods is a special case of what we have proved above. $\square$

It is interesting to observe that [23] also provides a low-rank variant of the Cayley subspace iteration algorithm: there, formulas for updating the factors of $X^{\mathsf {cay}}_{k}= Z^{\mathsf {cay}}_{k}(Y^{\mathsf {cay}}_{k})^{-1} (Z^{\mathsf {cay}}_{k})^*$, where $Z^{\mathsf {cay}}_{k}\in {\mathbb {C}}^{n \times pk}$ and $Y^{\mathsf {cay}}_{k}\in {\mathbb {C}}^{pk \times pk}$, are given. The contribution of [24] was to show that the same formulas can be derived from a control-theory point of view. The main difference in comparison to our RADI variant of the low-rank Riccati ADI iterations is that, in order to compute $Z^{\mathsf {cay}}_{k}$, one uses the matrix $(A^*+{\sigma _k}I)^{-1}$, instead of $(A^*-X_{k-1}G+{\sigma _k}I)^{-1}$, when computing $Z_k$. This way, the need for using the Sherman–Morrison–Woodbury formula is avoided. However, as a consequence, the matrix $Y^{\mathsf {cay}}_{k}$ looses the block-diagonal structure, and its update formula becomes much more involved. Also, it is very difficult to derive a version of the algorithm that would use real arithmetic. Another disadvantage is the computation of the residual: along with $Z^{\mathsf {cay}}_{k}$ and $Y^{\mathsf {cay}}_{k}$, one needs to maintain a QR-factorization of the matrix $[C^*\;\; A^*Z^{\mathsf {cay}}_{k}\;\; Z^{\mathsf {cay}}_{k}]$, which adds significant computational complexity to the algorithm.

Each of these different statements of the same Riccati ADI algorithm may contribute when studying theoretical properties of the iteration. For example, directly from our definition (12) of the RADI iteration it is obvious that

$$\begin{aligned} 0 \le X_1 \le X_2 \le \ldots \le X_k \le \ldots \le X. \end{aligned}$$

Also, the fact that the residual matrix $\mathscr {R}(X_k)$ is low-rank and its explicit factorization follows naturally from our approach. On the other hand, approaching the iteration from the control theory point of view as in [24] is more suitable for proving that the non-Blaschke condition for the shifts,

$$\begin{aligned} \sum _{k=1}^{\infty } \frac{{\text {Re}}\left( \sigma _k\right) }{1 + |\sigma _k|^2} = -\infty , \end{aligned}$$

is sufficient for achieving the convergence when *A* is stable, i.e.

$$\begin{aligned} \lim _{k \rightarrow \infty } X_k = X. \end{aligned}$$

We conclude this section by noting a relation between the Riccati ADI iteration and the rational Krylov subspace method [34]. It is easy to see that the RADI iteration also uses the rational Krylov subspaces as the basis for approximation. This fact also follows from the low-rank formulation for $X^{\mathsf {cay}}_{k}$ as given in [23], so we only state it here without proof.

### Proposition 2

For a matrix *M*, (block-)vector *v* and a tuple $\overrightarrow{\sigma _k} = (\sigma _1, \ldots , \sigma _k) \in \mathbb {C}_-^k$, let

$$\begin{aligned} \mathscr {K}(M, v, \overrightarrow{\sigma _k}) = {\text {span}}\{ (M+\sigma _1 I)^{-1}v, (M+\sigma _2 I)^{-1}v, \ldots , (M+\sigma _k I)^{-1}v \} \end{aligned}$$

denote the rational Krylov subspace generated by *M* and the initial vector *v*. Then the columns of $X_k$ belong to $\mathscr {K}(A^*, C^*, \overrightarrow{\sigma _k})$.

From the proposition we conclude the following: if $U_k$ contains a basis for the rational Krylov subspace $\mathscr {K}(A^*, C^*, \overrightarrow{\sigma _k})$, then both the approximation $X^{\mathsf {kry}}_{k}$ of the Riccati solution obtained by the rational Krylov subspace method and the approximation $X^{\mathsf {adi}}_{k}$ obtained by a Riccati ADI iteration satisfy

$$\begin{aligned} X^{\mathsf {kry}}_{k}= U_k Y^{\mathsf {kry}}_{k}U_k^*, \quad X^{\mathsf {adi}}_{k}= U_k Y^{\mathsf {adi}}_{k}U_k^*, \end{aligned}$$

for some matrices $Y^{\mathsf {kry}}_{k}, Y^{\mathsf {adi}}_{k}\in {\mathbb {C}}^{pk \times pk}$. The columns of both $X^{\mathsf {kry}}_{k}$ and $X^{\mathsf {adi}}_{k}$ belong to the same subspace, and the only difference between the methods is the choice of the linear combination of columns of $U_k$, i.e. the choice of the small matrix $Y_k$. The rational Krylov subspace method [34] generates its $Y^{\mathsf {kry}}_{k}$ by solving the projected Riccati equation, while the Riccati ADI methods do it via direct formulas such as the one in (12).

## Implementation aspects of the RADI algorithm

There are several issues with the iteration (12), stated as is, that should be addressed when designing an efficient computational routine: how to decide when the iterates $X_k$ have converged, how to solve linear systems with matrices $A^*- X_{k-1}G + \sigma _k I$, and how to minimize the usage of complex arithmetic. In this section we also discuss the various shift selection strategies.

### Computing the residual and the stopping criterion

Tracking the progress of the algorithm and deciding when the iterates have converged is very simple, and can be computed cheaply thanks to the expression $\Vert \mathscr {R}(X_k)\Vert = \Vert R_k R_k^*\Vert = \Vert R_k^*R_k\Vert .$ This is an advantage compared to the Cayley subspace iteration, where computing $\Vert \mathscr {R}(X_k)\Vert$ is more expensive because a low-rank factorization along the lines of Proposition 1 (b) is currently not known. The RADI iteration is stopped once the residual norm has decreased sufficiently relative to the initial residual norm $\Vert C C^*\Vert$ of the approximation $X_0=0$.

### Solving linear systems in RADI

During the iteration, one has to evaluate the expression $(A^*- X_{k-1}G + \sigma _k I)^{-1} R_{k-1}$. Here the matrix *A* is assumed to be sparse, while $X_{k-1}G = (X_{k-1}B) B^*$ is low-rank. There are different options on how to solve this linear system; if one wants to use a direct sparse solver, the initial expression can be adapted by using the Sherman–Morrison–Woodbury (SMW) formula [14]. We introduce the approximate feedback matrix $K_{k} := X_k B$ and update it during the RADI iteration: $K_{k} = K_{k-1} + (V_{k} {\tilde{Y}}_{k}^{-1}) (V_k^*B)$. Note that $V_k {\tilde{Y}}_k^{-1}$ also appears in the update of the residual factor, and that $V_k^*B$ appears in the computation of ${\tilde{Y}}_k$, so both have to be computed only once. The initial expression is rewritten as

$$\begin{aligned} (A^*- K_{k-1}B^*+ \sigma _k I)^{-1} R_{k-1}&=L_k+N_k(I_m-B^*N_k)^{-1}K_{k-1}^*L_k,\\ [L_k,N_k]&=(A^*+\sigma _kI)^{-1}[R_{k-1},K_{k-1}]. \end{aligned}$$

Thus, in each RADI step one needs to solve a linear system with the coefficient matrix $A^*+ \sigma _k I$ and $p+m$ right hand sides. A very similar technique is used in the low-rank Newton ADI solver for the Riccati equation [7, 9, 15, 29]. In the equivalent Cayley subspace iteration [23, 24], linear systems defined by $A^*+ \sigma _k I$ and only *p* right hand sides have to be solved, which makes their solution less expensive than their counterparts in RADI.
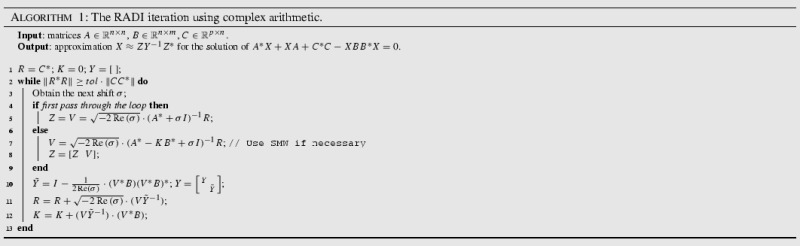


The RADI algorithm, implementing the techniques described above, is listed in Algorithm 1. Note that, if only the feedback matrix *K* is of interest, e.g. if the CARE arises from an optimal control problem, there is no need to store the whole low-rank factors *Z*, *Y* since Algorithm 1 requires only the latest blocks to continue. This is again similar to the low-rank Newton ADI solver [7], and not possible in the current version of the Cayley subspace iteration.

### Reducing the use of complex arithmetic

To increase the efficiency of Algorithm 1, we reduce the use of complex arithmetic. We do so by taking shift $\sigma _{k+1} = \overline{\sigma _k}$ immediately after the shift $\sigma _k \in {\mathbb {C}}\setminus {\mathbb {R}}$ has been used, and by merging these two consecutive RADI steps into a single one. This entire procedure will have only one operation involving complex matrices: a linear solve with the matrix $A^*- X_{k-1}G + \sigma _k I$ to compute $V_k$. There are two key observations to be made here. First, by modifying the iteration slightly, one can ensure that the matrices $K_{k+1}$, $R_{k+1}$, and $Y_{k+1}$ are real and can be computed by using real arithmetic only, as shown in the upcoming technical proposition. Second, there is no need to compute $V_{k+1}$ at all to proceed with the iteration: the next matrix $V_{k+2}$ will once again be computed by using the residual $R_{k+1}$, the same way as in Algorithm 1.

#### Proposition 3

Let $X_{k-1} = Z_{k-1} Y_{k-1}^{-1} Z_{k-1}^*\in {\mathbb {R}}^{n \times n}$ denote the Riccati approximate solution computed after $k-1$ RADI steps. Assume that $R_{k-1},~V_{k-1},~K_{k-1}$ are real and that $\sigma := \sigma _{k} = \overline{\sigma _{k+1}} \in {\mathbb {C}}\setminus {\mathbb {R}}$. Let:

$$\begin{aligned} V_{r}&= ({\text {Re}}\left( V_k\right) )^*B, \quad V_{i} = ({\text {Im}}\left( V_k\right) )^*B, \\ F_1&= \left[ \begin{array}{c} -{\text {Re}}\left( \sigma \right) V_{r} - {\text {Im}}\left( \sigma \right) V_{i} \\ {\text {Im}}\left( \sigma \right) V_{r} - {\text {Re}}\left( \sigma \right) V_{i} \end{array}\right] , \quad F_2 = \left[ \begin{array}{c} V_{r} \\ V_{i} \end{array}\right] , \quad F_3 = \left[ \begin{array}{c} {\text {Im}}\left( \sigma \right) I_p \\ {\text {Re}}\left( \sigma \right) I_p \end{array}\right] . \end{aligned}$$

Then $$X_{k+1} = Z_{k+1} Y_{k+1}^{-1} Z_{k+1}^*$$, where:

$$\begin{aligned} Z_{k+1}&= [Z_{k-1} \;\; {\text {Re}}\left( V_k\right) \;\; {\text {Im}}\left( V_k\right) ], \\ Y_{k+1}&= \left[ \begin{array}{cc} Y_{k-1} &{} \\ &{} {\hat{Y}}_{k+1} \end{array}\right] , \\ {\hat{Y}}_{k+1}&= \left[ \begin{array}{cc} I_p &{} \\ &{} 1/2 I_p \end{array}\right] - \frac{1}{4|\sigma |^2 {\text {Re}}\left( \sigma \right) } F_1 F_1^*- \frac{1}{4{\text {Re}}\left( \sigma \right) } F_2 F_2^*- \frac{1}{2|\sigma |^2} F_3 F_3^*. \end{aligned}$$

The residual factor $R_{k+1}$ and the matrix $K_{k+1}$ can be computed as

$$\begin{aligned} R_{k+1}&= R_{k-1} + \sqrt{-2{\text {Re}}\left( \sigma \right) } \left( [{\text {Re}}\left( V_k\right) \;\; {\text {Im}}\left( V_k\right) ] {\hat{Y}}_{k+1}^{-1} \right) (:, 1:p), \\ K_{k+1}&= K_{k-1} + [{\text {Re}}\left( V_k\right) \;\; {\text {Im}}\left( V_k\right) ] {\hat{Y}}_{k+1}^{-1} \left[ \begin{array}{c} V_{r} \\ V_{i} \end{array}\right] . \end{aligned}$$

#### Proof

The basic idea of the proof is similar to [5, Theorem 1]; however, there is a major complication involving the matrix *Y*, which in the Lyapunov case is simply equal to the identity. Due to the technical complexity of the proof, we only display key intermediate results. To simplify notation, we use indices 0, 1, 2 instead of $k-1$, *k*, $k+1$, respectively.

We start by taking the imaginary part of the defining relation for $R_0^{(2)}=R_0$ in Lemma 1:

$$\begin{aligned} 0 = (A^*- X_0 G + {\text {Re}}\left( \sigma \right) I) \cdot {\text {Im}}\left( V_1\right) + {\text {Im}}\left( \sigma \right) \cdot {\text {Re}}\left( V_1\right) ; \end{aligned}$$

thus

$$\begin{aligned} V_1 = {\text {Re}}\left( V_1\right) + \mathsf {i}{\text {Im}}\left( V_1\right) = \frac{-1}{{\text {Im}}\left( \sigma \right) } \underbrace{(A^*- X_0 G + \overline{\sigma } I)}_{A_0} {\text {Im}}\left( V_1\right) . \end{aligned}$$

We use this and the Sherman–Morrison–Woodbury formula to compute $V_2$:

24 $$\begin{aligned} V_2&= \sqrt{-2{\text {Re}}\left( \sigma \right) } (A^*- X_1 G + \overline{\sigma } I)^{-1} R_1 \nonumber \\&= \sqrt{-2{\text {Re}}\left( \sigma \right) } (A^*- X_1 G + \overline{\sigma } I)^{-1} \cdot \frac{1}{\sqrt{-2{\text {Re}}\left( \sigma \right) }} \cdot (A^*- X_1 G - \overline{\sigma }I ) V_1\nonumber \\&= V_1 - 2\overline{\sigma } (A^*- X_1 G + \overline{\sigma }I)^{-1} V_1 \nonumber \\&= V_1 - 2\overline{\sigma } \left( A_0 - V_1 {\tilde{Y}}_1^{-1} \left( V_1^*G\right) \right) ^{-1} V_1 \nonumber \\&= V_1 - 2\overline{\sigma } \left( A_0^{-1}V_1 + A_0^{-1} V_1 \left( {\tilde{Y}}_1 - \left( V_1^*G\right) A_0^{-1}V_1\right) ^{-1} \left( V_1^*G\right) A_0^{-1}V_1 \right) \nonumber \\&= V_1 + 2\overline{\sigma } {\text {Im}}\left( V_1\right) \left( \underbrace{{\text {Im}}\left( \sigma \right) {\tilde{Y}}_1 + \left( V_1^*B\right) V_{i}^*}_{S}\right) ^{-1} {\tilde{Y}}_1; \end{aligned}$$

where we used ${\text {Im}}\left( \sigma \right) A_0^{-1} V_1 = -{\text {Im}}\left( V_1\right)$ to obtain the last line. Next,

$$\begin{aligned} X_2&= X_0 + \left[ {\begin{matrix} V_1&V_2 \end{matrix}}\right] \left[ {\begin{matrix} {\tilde{Y}}_1^{-1} &{} \\ &{} {\tilde{Y}}_2^{-1} \end{matrix}}\right] \left[ {\begin{matrix} V_1&V_2 \end{matrix}}\right] ^*\\&= X_0 + \left[ {\begin{matrix} {\text {Re}}\left( V_1\right)&{\text {Im}}\left( V_1\right) \end{matrix}}\right] \underbrace{\left[ {\begin{matrix} I &{} I \\ \mathsf {i}I &{} \mathsf {i}I + 2\overline{\sigma } S^{-1} {\tilde{Y}}_1 \end{matrix}}\right] }_T \left[ {\begin{matrix} {\tilde{Y}}_1^{-1} &{} \\ &{} {\tilde{Y}}_2^{-1} \end{matrix}}\right] \\&\quad \,\times \left( \left[ {\begin{matrix} {\text {Re}}\left( V_1\right)&{\text {Im}}\left( V_1\right) \end{matrix}}\right] \left[ {\begin{matrix} I &{} I \\ \mathsf {i}I &{} \mathsf {i}I + 2\overline{\sigma } S^{-1} {\tilde{Y}}_1 \end{matrix}}\right] \right) ^*\\&= X_0 + \left[ {\begin{matrix} {\text {Re}}\left( V_1\right)&{\text {Im}}\left( V_1\right) \end{matrix}}\right] \big ( \underbrace{T^{-*} \left[ {\begin{matrix} {\tilde{Y}}_1 &{} \\ &{} {\tilde{Y}}_2 \end{matrix}}\right] T^{-1}}_{\hat{Y}_2} \big )^{-1} \left[ {\begin{matrix} {\text {Re}}\left( V_1\right)&{\text {Im}}\left( V_1\right) \end{matrix}}\right] ^*. \end{aligned}$$

We first compute $T^{-1}$ by using the SMW formula once again:

$$\begin{aligned} T^{-1}&= \left( \left[ {\begin{matrix} &{} I \\ \mathsf {i}I &{} \mathsf {i}I \end{matrix}}\right] + \left[ {\begin{matrix} I &{} \\ &{} S^{-1} \end{matrix}}\right] \left[ {\begin{matrix} I &{} \\ &{} 2\overline{\sigma }{\tilde{Y}}_1 \end{matrix}}\right] \right) ^{-1} \\&= \left[ \begin{array}{cc} I + \frac{\mathsf {i}}{2\overline{\sigma }} {\tilde{Y}}_1^{-1}S &{} \frac{-1}{2\overline{\sigma }}{\tilde{Y}}_1^{-1} S \\ \frac{-\mathsf {i}}{2\overline{\sigma }} {\tilde{Y}}_1^{-1}S &{} \frac{1}{2\overline{\sigma }} {\tilde{Y}}_1^{-1}S\end{array}\right] , \end{aligned}$$

and, applying the congruence transformation with $T^{-1}$ to $$\left[ {\begin{matrix} {\tilde{Y}}_1 &{} \\ &{} {\tilde{Y}}_2 \end{matrix}}\right] $$ yields

$$\begin{aligned}&{\hat{Y}}_2 =\\&\left[ \begin{array}{cc} {\tilde{Y}}_1 + \frac{\mathsf {i}}{2\overline{\sigma }} S - \frac{\mathsf {i}}{2{\sigma }} S^*+ \frac{1}{4|\sigma |^2} S^*{\tilde{Y}}_1^{-1}S + \frac{1}{4|\sigma |^2} S^*{\tilde{Y}}_1^{-1} {\tilde{Y}}_2 {\tilde{Y}}_1^{-1} S ~~~~&{}~~~\frac{-1}{2\overline{\sigma }} S + \frac{\mathsf {i}}{4|\sigma |^2} S^*{\tilde{Y}}_1^{-1}S + \frac{\mathsf {i}}{4|\sigma |^2}S^*{\tilde{Y}}_1^{-1} {\tilde{Y}}_2 {\tilde{Y}}_1^{-1} S \\ \frac{-1}{2{\sigma }} S^*- \frac{\mathsf {i}}{4|\sigma |^2} S^*{\tilde{Y}}_1^{-1}S - \frac{\mathsf {i}}{4|\sigma |^2} S^*{\tilde{Y}}_1^{-1} {\tilde{Y}}_2 {\tilde{Y}}_1^{-1} S &{} \frac{1}{4|\sigma |^2} S^*{\tilde{Y}}_1^{-1}S + \frac{1}{4|\sigma |^2} S^*{\tilde{Y}}_1^{-1} {\tilde{Y}}_2 {\tilde{Y}}_1^{-1} S \end{array}\right] . \end{aligned}$$

By using (24), it is easy to show

$$\begin{aligned} {\tilde{Y}}_2=&\, I - \frac{1}{2{\text {Re}}\left( \sigma \right) } \left( V_2^*B\right) \left( V_2^*B\right) ^*\\ =&\, -{\tilde{Y}}_1 - \frac{1}{2{\text {Re}}\left( \sigma \right) } \\&\times \left( -2\sigma {\text {Im}}\left( \sigma \right) {\tilde{Y}}_1S^{-*}{\tilde{Y}}_1 - 2\overline{\sigma }{\text {Im}}\left( \sigma \right) {\tilde{Y}}_1 S^{-1}{\tilde{Y}}_1 +4|\sigma |^2 {\tilde{Y}}_1S^{-*} V_{i} V_{i}^*S^{-1} {\tilde{Y}}_1 \right) . \end{aligned}$$

Inserting this into the formula for ${\hat{Y}}_2$, all terms containing inverses of ${\tilde{Y}}_1$ and *S* cancel out. By rearranging the terms that do appear in the formula, we get the expression from the claim of the proposition.

Deriving the formulae for $R_{2}$ and $K_{2}$ is straightforward:

$$\begin{aligned} K_{2}&= K_{0} + \left[ {\begin{matrix} V_1&V_2 \end{matrix}}\right] \left[ {\begin{matrix} {\tilde{Y}}_1^{-1} &{} \\ &{} {\tilde{Y}}_2^{-1} \end{matrix}}\right] \left[ {\begin{matrix} V_1&V_2 \end{matrix}}\right] ^*B\\&= K_0 + \left( \left[ {\begin{matrix} {\text {Re}}\left( V_1\right)&{\text {Im}}\left( V_1\right) \end{matrix}}\right] {\hat{Y}}_2^{-1} \right) \left( \left[ {\begin{matrix} {\text {Re}}\left( V_1\right)&{\text {Im}}\left( V_1\right) \end{matrix}}\right] ^*B \right) , \\ R_2&= R_0 + \sqrt{-2{\text {Re}}\left( \sigma \right) } \left[ {\begin{matrix} V_1&V_2 \end{matrix}}\right] \left[ {\begin{matrix} {\tilde{Y}}_1^{-1} \\ {\tilde{Y}}_2^{-1} \end{matrix}}\right] \\&= R_0 + \sqrt{-2{\text {Re}}\left( \sigma \right) } \left[ {\begin{matrix} {\text {Re}}\left( V_1\right)&{\text {Im}}\left( V_1\right) \end{matrix}}\right] \left( T \left[ {\begin{matrix} {\tilde{Y}}_1^{-1} \\ {\tilde{Y}}_2^{-1} \end{matrix}}\right] \right) \\&= R_0 + \sqrt{-2{\text {Re}}\left( \sigma \right) } \left[ {\begin{matrix} {\text {Re}}\left( V_1\right)&{\text {Im}}\left( V_1\right) \end{matrix}}\right] {\hat{Y}}_2^{-1} (:, 1:p), \end{aligned}$$

where the last line is due to the structure of the matrix *T*. Since the matrix ${\hat{Y}}_2$ is real, so are $K_2$ and $R_2$. $\square$


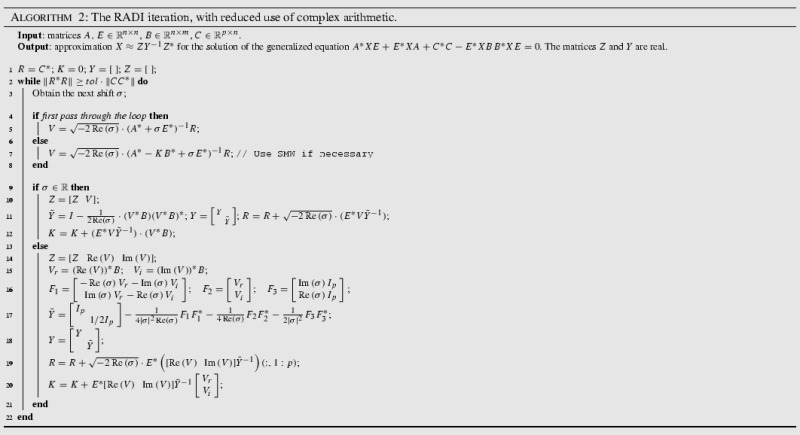


### RADI iteration for the generalized Riccati equation

Before we state the final implementation, we shall briefly mention the adaptation of the RADI algorithm for handling generalized Riccati equations

25 $$\begin{aligned} A^*XE + E^*X A + Q - E^*XGXE = 0. \end{aligned}$$

Multiplying (25) by $E^{-*}$ from the left and by $E^{-1}$ from the right leads to

26 $$\begin{aligned} (AE^{-1})^*X + X (AE^{-1}) + E^{-*} C^*C E^{-1} - X BB^*X = 0. \end{aligned}$$

The generalized RADI algorithm is then easily derived by running ordinary RADI iterations for the Riccati equation (26), and deploying standard rearrangements to lower the computational expense (such as solving systems with the matrix $A^*+ \sigma E^*$ instead of $(AE^{-1})^*+ \sigma I$). Algorithm 2 shows the final implementation, taking into account Proposition 3 and the handling of generalized Eq. (25).

### Shift selection

The problem of choosing the shifts in order to accelerate the convergence of the Riccati ADI iteration is very similar to the one for the Lyapunov ADI method. Thus we apply and discuss the techniques presented in [3, 6, 27, 30] in the context of the Riccati equation, and compare them in several numerical experiments. It appears natural to employ the heuristic Penzl shifts [27]. There, a small number of approximate eigenvalues of *A* are generated. From this set the values which lead to the smallest magnitude of the rational function associated to the ADI iteration are selected in a heuristical manner. Simoncini and Lin [23] have shown that the convergence of the Riccati ADI iteration is related to a rational function built from the stable eigenvalues of $\mathscr {H}$. This suggests to carry out the Penzl approach, but to use approximate eigenvalues for the Hamiltonian matrix $\mathscr {H}$ instead of *A*. Note that, due to the low rank of *Q* and *G*, we can expect that most of the eigenvalues of *A* are close to the eigenvalues of $\mathscr {H}$, see the discussion in [3]. Thus in many cases the Penzl shifts generated by *A* should suffice as well. Penzl shifts require significant preprocessing computation: in order to approximate the eigenvalues of $M=A$ or $M=\mathscr {H}$, one has to build Krylov subspaces with matrices *M* and $M^{-1}$. All the shifts are computed in this preprocessing stage, and then simply cycled during the RADI iteration. Here, we will mainly focus on alternative approaches generating each shift just before it is used. This way we hope to compute a shift which will better adapt to the current stage of the algorithm.

#### Residual Hamiltonian shifts

One such approach is motivated by Theorem 1 and the discussion about shift selection in [3]. The Hamiltonian matrix

$$\begin{aligned} \tilde{\mathscr {H}} = \left[ \begin{array}{cc} {\tilde{A}} &{} G \\ {\tilde{C}}^*{\tilde{C}} &{} -{\tilde{A}}^*\end{array}\right] \end{aligned}$$

is associated to the residual equation (10), where $\varXi = X_k$ is the approximation after *k* steps of the RADI iteration. If $$(\lambda , \left[ {\begin{matrix} r \\ q \end{matrix}}\right] )$$ is a stable eigenpair of $\tilde{\mathscr {H}}$, and $\sigma _{k+1} = \lambda$ is used as the shift, then

$$\begin{aligned} X_k \le X_{k+1} = X_k - q (q^*r)^{-1} q^*\le X. \end{aligned}$$

In order to converge as fast as possible to *X*, it is better to choose such an eigenvalue $\lambda$ for which the update is largest, i.e. the one that maximizes $\Vert q (q^*r)^{-1} q^*\Vert$. Note that as the RADI iteration progresses and the residual matrix $\mathscr {R}(\varXi )={\tilde{C}}^*{\tilde{C}}$ converges to zero, the structure of eigenvectors of $\tilde{\mathscr {H}}$ that belong to its stable eigenvalues is such that $\Vert q\Vert$ becomes smaller and smaller. Thus, one can further simplify the shift optimality condition, and use the eigenvalue $\lambda$ such that the corresponding *q* has the largest norm—this is also in line with the discussion in [3].

However, in practice it is computationally very expensive to determine such an eigenvalue, since the matrix $\tilde{\mathscr {H}}$ is of order 2*n*. We can approximate its eigenpairs through projection onto some subspace. If *U* is an orthonormal basis of the chosen subspace, then

$$\begin{aligned}&(U^*{\tilde{A}} U)^*{\tilde{X}}^{\mathsf {proj}}+ {\tilde{X}}^{\mathsf {proj}}(U^*{\tilde{A}} U) + (U^*{\tilde{C}}^*) (U^*{\tilde{C}}^*)^*\\&\quad - {\tilde{X}}^{\mathsf {proj}}(U^*B) (U^*B)^*{\tilde{X}}^{\mathsf {proj}}= 0 \end{aligned}$$

is the projected residual Riccati equation with the associated Hamiltonian matrix

27 $$\begin{aligned} \tilde{\mathscr {H}}^{\mathsf {proj}}= \left[ \begin{array}{cc} U^*{\tilde{A}} U &{} (U^*B) (U^*B)^*\\ (U^*{\tilde{C}}^*) (U^*{\tilde{C}}^*)^*&{} -(U^*{\tilde{A}} U)^*\end{array}\right] . \end{aligned}$$

Approximate eigenpairs of $\tilde{\mathscr {H}}$ are $$({\hat{\lambda }}, \left[ {\begin{matrix} U {\hat{r}} \\ U {\hat{q}} \end{matrix}}\right] )$$, where $$({\hat{\lambda }}, \left[ {\begin{matrix} {\hat{r}} \\ {\hat{q}} \end{matrix}}\right] )$$ are eigenpairs of $\tilde{\mathscr {H}}^{\mathsf {proj}}$. Thus, a reasonable choice for the next shift is such $\hat{\lambda }$, for which $\Vert U \hat{q}\Vert = \Vert \hat{q}\Vert$ is the largest.

We still have to define the subspace ${\text {span}}\{ U \}$. One option is to use $V_k$ (or, equivalently, the last *p* columns of the matrix $Z_k$), which works very well in practice unless $p=1$. When $p=1$, all the generated shifts are real, which can make the convergence slow in some cases. Then it is better to choose the last $\ell$ columns of the matrix $Z_k$; usually already $\ell =2$ or $\ell =5$ or a small multiple of *p* will suffice. An ultimate option is to use the entire $Z_k$, which we denote as $\ell =\infty$. This is obviously more computationally demanding, but it provides fast convergence in all cases we tested.

#### Residual minimizing shifts

The two successive residual factors are connected via the formula

$$\begin{aligned} R_{k+1} = (A^*- X_{k+1}G - \overline{\sigma _{k+1}}I) (A^*- X_k G + \sigma _{k+1} I)^{-1} R_k. \end{aligned}$$

Our goal is to choose the shifts so that the residual drops to zero as quickly as possible. Locally, once $X_k$ is computed, this goal is achieved by choosing $\sigma _{k+1}\in \mathbb {C}_-$ so that $\Vert R_{k+1}\Vert$ is minimized. This concept was proposed in [6] for the low-rank Lyapunov and Sylvester ADI methods and refined later in [18]. In complete analogy, we define a rational function *f* in the variable $\sigma$ by

28 $$\begin{aligned} f(\sigma ) := \Vert (A^*- X_{k+1}(\sigma ) G - \overline{\sigma } I) (A^*- X_k G + \sigma I)^{-1} R_k\Vert ^2, \end{aligned}$$

and wish to find

$$\begin{aligned} {\text {argmin}}_{\sigma \in \mathbb {C}_-} f(\sigma ); \end{aligned}$$

note that $X_{k+1}(\sigma ) = X_k + V_{k+1}(\sigma ){\tilde{Y}}_{k+1}^{-1}(\sigma ) V_{k+1}^*(\sigma )$ is also a function of $\sigma$. Since *f* involves large matrices, we once again project the entire equation to a chosen subspace *U*, and solve the optimization problem defined by the matrices of the projected problem. The optimization problem is solved numerically. Efficient optimization solvers use the gradient of the function *f*; after a laborious computation one can obtain an explicit formula for the case $p=1$:

$$\begin{aligned}&\nabla f(\sigma _R, \sigma _I)\\&\quad = \left[ \begin{array}{c} 2{\text {Re}}\left( R_{k+1}^*\cdot \left( \left( \frac{1}{\sigma _R} I - {\tilde{A}}^{-1} - \frac{1}{2\sigma _R} \left( X_{k+1}G{\tilde{A}}^{-1} + X_{k+1}{\tilde{A}}^{-*}G\right) \right) \varDelta \right) \right) \\ -2{\text {Im}}\left( R_{k+1}^*\cdot \left( \left( -{\tilde{A}}^{-1} - \frac{1}{2\sigma _R} \left( X_{k+1} G {\tilde{A}}^{-1} - X_{k+1}{\tilde{A}}^{-*}G\right) \right) \varDelta \right) \right) \end{array}\right] . \end{aligned}$$

Here $$\sigma = \sigma _R + \mathsf {i}\sigma _I$$, $${\tilde{A}} = A^*- X_k G - \sigma I$$, $$X_{k+1} = X_{k+1}(\sigma )$$, $$R_{k+1} = R_{k+1}(\sigma )$$, and $$\varDelta = R_{k+1}(\sigma ) - R_k$$.

For $p>1$, a similar formula can be derived, but one should note that the function *f* is not necessarily differentiable at every point $\sigma$, see, e.g., [26]. Thus, a numerically more reliable heuristic [18] is to artificially reduce the problem once again to the case $p=1$. This can be done in the following way: let *v* denote the right singular vector corresponding to the largest singular value of the matrix $R_k$. Then $R_k\in {\mathbb {C}}^{n \times p}$ in (28) is replaced by the vector $R_k v \in {\mathbb {C}}^{n}$. Since numerical optimization algorithms usually require a starting point for the optimization, the two shift generating approaches may be combined: the residual Hamiltonian shift can be used as the starting point in the optimization for the second approach. However, from our numerical experience we conclude that the additional computational effort invested in the post-optimization of the residual Hamiltonian shifts often does not contribute to the convergence. The main difficulty is the choice of an adequate subspace *U* such that the projected objective function approximates (28) well enough. This issue requires futher investigation. The rationale is given in the following example.

##### Example 1

Consider the Riccati equation given in Example 5.2 of [32]: the matrix *A* is obtained by the centered finite difference discretization of the differential equation

$$\begin{aligned} \partial _t u = \varDelta u - 10 x \partial _x u - 1000 y \partial _y u - 10 \partial _z u + b(x, y)f(t), \end{aligned}$$

on a unit cube with 22 nodes in each direction. Thus *A* is of order $n=10648$; the matrices $B \in {\mathbb {R}}^{n \times m}$ and $C \in {\mathbb {R}}^{p \times n}$ are generated at random, and in this example we set $m=p=1$ and $B=C^*$.

Suppose that 13 RADI iterations have already been computed, and that we need to compute a shift to be used in the 14th iteration. Let the matrix *U* contain an orthonormal basis for $Z_{13}$.

**Fig. 1:**
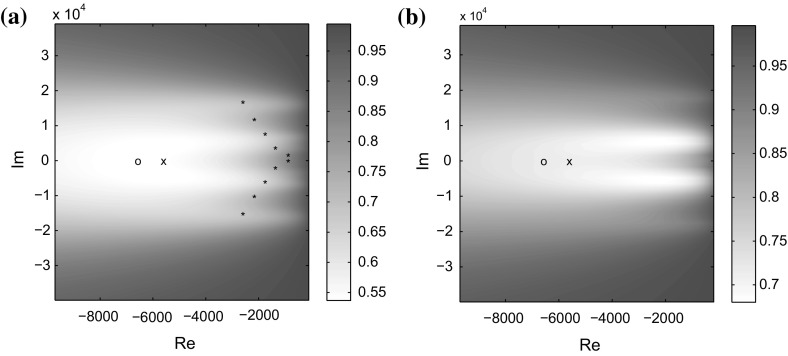
Each point $\sigma$ of the complex plane is colored according to residual reduction obtained when $\sigma$ is taken as the shift in the 14th iteration of RADI. **a** Ratios $\rho ^{\mathsf {proj}}(\sigma )=\Vert R^{\mathsf {proj}}_{14}(\sigma )\Vert / \Vert R^{\mathsf {proj}}_{13}\Vert$ for the projected equation of dimension 13. **b** Ratios $\rho (\sigma )=\Vert R_{14}(\sigma )\Vert / \Vert R_{13}\Vert$ for the original equation of dimension 10648

Figure 1a shows a region of the complex plane; stars are at locations of the stable eigenvalues of the projected Hamiltonian matrix (27). The one eigenvalue chosen as the residual Hamiltonian shift $\sigma _{\mathsf {ham}}$ is shown as ‘x’. The residual minimizing shift $\sigma _{\mathsf {opt}}$ is shown as ‘o’. Each point $\sigma$ of the complex plane is colored according to the ratio $\rho ^{\mathsf {proj}}(\sigma )=\Vert R^{\mathsf {proj}}_{14}(\sigma )\Vert / \Vert R^{\mathsf {proj}}_{13}\Vert$, where $R^{\mathsf {proj}}$ is the residual for the *projected* Riccati equation. The ratio $\rho ^{\mathsf {proj}}(\sigma _{\mathsf {ham}}) \approx 0.54297$ is not far from the optimal ratio $\rho ^{\mathsf {proj}}(\sigma _{\mathsf {opt}}) \approx 0.53926$.

On the other hand, Fig. 1b shows the complex plane colored according to ratios for the original system of order 10648, $\rho (\sigma ) = \Vert R_{14}(\sigma )\Vert / \Vert R_{13}\Vert$. Neither of the values $\rho (\sigma _{\mathsf {ham}}) \approx 0.71510$ and $\rho (\sigma _{\mathsf {opt}}) \approx 0.71981$ is optimal, but they both offer a reasonable reduction of the residual norm in the next step. In this case, $\sigma _{\mathsf {ham}}$ turns out even to give a slightly better residual reduction for the original equation than $\sigma _{\mathsf {opt}}$, making the extra effort in running the numerical optimization algorithm futile.

## Numerical experiments

In this section we show a number of numerical examples, with several objectives in mind. First, our goal is to compare different low-rank implementations of the Riccati ADI algorithm mentioned in this paper: the low-rank qADI proposed in [37, 38], the Cayley transformed subspace iteration [23, 24], and the complex and real variants of the RADI iteration (12). Second, we compare performance of the RADI approach against other methods for solving large-scale Riccati equations, namely the rational Krylov subspace method (RKSM) [34], the extended block Arnoldi (EBA) method [16, 32], and the Newton-ADI algorithm [7, 9, 15].

Finally, we discuss various shift strategies for the RADI iteration described in the previous section.

The numerical experiments are run on a desktop computer with a four-core Intel Core i5-4690K processor and 16GB RAM. All algorithms and testing routines are implemented and executed in MATLAB R2014a, running on Microsoft Windows 8.1.

### Example 2

Consider again the Riccati benchmark CUBE from Example 1. We use three versions of this example: the previous setting with $n=10648$, $m=p=1$ and $m=p=10$, and later on a finer discretization with $n=74088$ and $m=10$, $p=1$.

Table 1 collects timings in seconds for four different low-rank implementations of the Riccati ADI algorithm. The table shows only the time needed to run 80 iteration steps; time spent for computation of the shifts used by all four variants is not included (in this case, 20 precomputed Penzl shifts were used). All four variants compute exactly the same iterates, as we have proved in Theorem 2.

**Table 1:** Results obtained with different implementations for CUBE with $n=10648$

Implementation	Time, $m,p = 1$	Time, $m,p = 10$
Wong and Balakrishnan [37, 38]	127.61	750.89
Cayley subspace iteration [23, 24]	21.67	167.02
RADI—Algorithm 1	21.51	51.92
RADI—Algorithm 2	11.14	26.35

Clearly, the real variant of iteration (12), implemented as in Algorithm 2, outperforms all the others.^1^ Thus we use this implementation in the remaining numerical experiments. The RADI algorithms mostly obtain the advantage over the Cayley subspace iteration because of the cheap computation of the residual norm. In the latter algorithm, costly orthogonalization procedures are required for this task, and after some point these compensate the computational gains from the easier linear systems (cf. Sect. 4.2). Also, the times for the algorithm of Wong and Balakrishnan shown in the table do not include the (very costly) computation of the residuals at all, so their actual execution times are even higher.

Next, we compare various shift strategies for RADI, as well as EBA, RKSM, and Newton-ADI algorithms. For RADI, we have the following strategies:

- 20 precomputed Penzl shifts (“RADI—Penzl”) generated by using the Krylov subspaces of dimensions 40 with matrices *A* and $A^{-1}$;
- residual Hamiltonian shifts (“RADI—Ham”), with $\ell =2p$, $\ell =6p$, and $\ell =\infty$;
- residual minimizing shifts (“RADI—Ham + Opt”), with $\ell =2p$, $\ell =6p$, and $\ell =\infty$.

For the RKSM, we have implemented the algorithm so that the use of complex arithmetic is minimized by merging two consecutive steps with complex conjugate shifts [28]. We use the adaptive shift strategy, implemented as described in [13]. EBA and RKSM require the solution of a projected CARE which can become expensive if $p>1$. Hence, this small scale solution is carried out only periodically in every 5th or every 10th step—in the results, we display the variant that was faster.

**Table 2:** Times spend in different subtasks in the RADI iteration and RKSM for CUBE with $m=p=10$

Method	Subtask	Time
RADI—Penzl: 135 iterations	Precompute shifts	5.31
	Solve linear systems	43.24
	Total	49.19
RADI—Ham, $\ell =2p$: 139 iterations	Solve linear systems	46.42
	Compute shifts dynamically	1.76
	Total	48.79
RADI—Ham, $\ell =6p$: 100 iterations	Solve linear systems	32.73
	Compute shifts dynamically	2.75
	Total	35.92
RADI—Ham, $\ell =\infty$: 74 iterations	Solve linear systems	24.74
	Compute shifts dynamically	32.44
	Total	57.51
RKSM—adaptive: 79 iterations	Solve linear systems	18.45
	Orthogonalization	4.14
	Compute shifts dynamically	12.02
	Solve projected equations	15.98
	Total	53.82

**Fig. 2:**
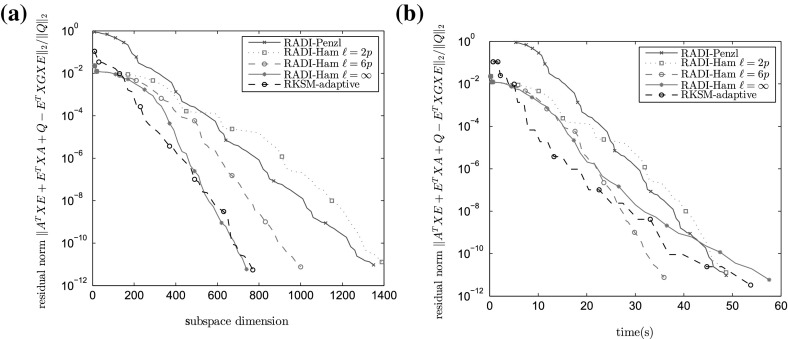
Algorithm performances for benchmark CUBE ($n=10648, m=p=10$). **a** Relative residual versus the subspace dimension used by an algorithm. **b** Relative residual versus time

**Table 3:** Results of the numerical experiments

Example	Method	No. iterations	Final subspace dim.	Time
CUBE $n=10648, \; m=p=1$	RADI—Penzl	97	97	18.96
	RADI—Ham, $\ell =2p$	119	119	17.10
	RADI—Ham, $\ell =6p$	99	99	14.15
	RADI—Ham, $\ell =\infty$	75	**75**	11.60
	RADI—Ham + Opt, $\ell =2p$	122	122	17.87
	RADI—Ham + Opt, $\ell =6p$	103	103	16.70
	RADI—Ham + Opt, $\ell =\infty$	108	108	18.44
	RKSM—adaptive	83	83	14.80
	EBA	111	222	**6**.**23**
	Newton-ADI	2 outer, 296 inner	192	42.11
CUBE $n=10648, \; m=p=10$	RADI—Penzl	135	1350	49.19
	RADI—Ham, $\ell =2p$	139	1390	48.79
	RADI—Ham, $\ell =6p$	100	1000	35.92
	RADI—Ham, $\ell =\infty$	74	**740**	57.51
	RADI—Ham + Opt, $\ell =2p$	87	870	**30**.**59**
	RADI—Ham + Opt, $\ell =6p$	90	900	33.20
	RADI—Ham + Opt, $\ell =\infty$	90	900	119.55
	RKSM—adaptive	79	790	53.82
	EBA	91	1820	230.57
	Newton-ADI	2 outer, 202 inner	1960	75.60
CUBE $n=74088, \; m=10, \; p=1$	RADI—Penzl	139	139	1048.60
	RADI—Ham, $\ell =2p$	97	97	617.62
	RADI—Ham, $\ell =6p$	81	81	506.37
	RADI—Ham, $\ell =\infty$	72	72	446.64
	RADI—Ham + Opt, $\ell =2p$	101	101	621.38
	RADI—Ham + Opt, $\ell =6p$	93	93	571.34
	RADI—Ham + Opt, $\ell =\infty$	63	**63**	387.43
	RKSM—adaptive	73	73	338.78
	EBA	81	162	**30**.**45**
	Newton-ADI	2 outer, 288 inner	968	1546.29
CHIP $n=20082, \; m=1, \; p=5$	RADI—Penzl	33	165	51.57
	RADI—Ham, $\ell =2p$	36	180	30.32
	RADI—Ham, $\ell =6p$	29	145	24.36
	RADI—Ham, $\ell =\infty$	26	130	22.64
	RADI—Ham + Opt, $\ell =2p$	29	145	23.97
	RADI—Ham + Opt, $\ell =6p$	26	130	22.26
	RADI—Ham + Opt, $\ell =\infty$	25	**125**	22.33
	RKSM—adaptive	26	130	23.33
	EBA	26	260	**6**.**69**
	Newton-ADI	2 outer, 64 inner	204	54.04
IFISS $n=66049, \; m=p=5$	RADI—Penzl	>50	>250	
	RADI—Ham, $\ell =2p$	22	110	17.21
	RADI—Ham, $\ell =6p$	19	**95**	15.37
	RADI—Ham, $\ell =\infty$	20	100	17.46
	RADI—Ham + Opt, $\ell =2p$	27	135	21.12
	RADI—Ham + Opt, $\ell =6p$		Did not converge	
	RADI—Ham + Opt, $\ell =\infty$		Did not converge	
	RKSM—adaptive	26	130	22.28
	EBA	11	110	**9**.**26**
	Newton-ADI	2 outer, 46 inner	250	38.05
RAIL $n=317377, \; m=7, \; p=6$	RADI—Penzl	66	396	182.60
	RADI—Ham, $\ell =2p$	49	294	131.34
	RADI—Ham, $\ell =6p$	43	258	127.11
	RADI—Ham, $\ell =\infty$	46	276	197.06
	RADI—Ham + Opt, $\ell =2p$	46	276	124.13
	RADI—Ham + Opt, $\ell =6p$	40	240	**120**.**04**
	RADI—Ham + Opt, $\ell =\infty$	39	**234**	158.89
	RKSM—adaptive	41	246	188.60
	EBA	91	1092	916.21
	Newton-ADI	1 outer, 62 inner	372	279.90
LUNG $n=109460, \; m=p=10$	RADI—Penzl		Did not converge	
	RADI—Ham, $\ell =2p$	31	310	30.03
	RADI—Ham, $\ell =6p$	28	280	30.22
	RADI—Ham, $\ell =\infty$	26	260	34.83
	RADI—Ham + Opt, $\ell =2p$	25	250	22.33
	RADI—Ham + Opt, $\ell =6p$	17	**170**	**17**.**74**
	RADI—Ham + Opt, $\ell =\infty$	17	**170**	19.02
	RKSM—adaptive	61	610	114.22
	EBA		Did not converge	
	Newton-ADI		Did not converge	

Numbers in bold indicate the smallest subspace dimensions and execution times

For all methods, the threshold for declaring convergence is reached once the relative residual is less than $tol=10^{-11}$. A summary of the results for all the different methods and strategies is shown in Table 3. The column “final subspace dimension” displays the number of columns of the matrix *Z*, where $X\approx ZZ^*$ is the final computed approximation. Dividing this number by *p* (for EBA, by 2*p*), we obtain the number of iterations used in a particular method. Just for the sake of completeness, we have also included a variant of the Newton-ADI algorithm [7] with Galerkin projection [9]. Without the Galerkin projection, the Newton-ADI algorithm could not compete with the other methods. The recent developments from [15], which make the Newton-ADI algorithm more competitive, are beyond the scope of this study.

It is interesting to analyze the timing breakdown for RADI and RKSM methods. These timings are listed in Table 2 for the CUBE example with $m=p=10$ where a significant amount of time is spent for tasks other than solving linear systems.

As $\ell$ increases, the cost of computing shifts in RADI increases as well—the projection subspace gets larger, and more effort is needed to orthogonalize its basis and compute the eigenvalue decomposition of the projected Hamiltonian matrix. This effort is, in the CUBE benchmark, awarded by a decrease in the number of iterations. However, there is a trade-off here: the extra computation does outweigh the saving in the number of iterations for sufficiently large $\ell$. Convergence history for CUBE is plotted in Fig. 2; to reduce the clutter, only the selected few methods are shown.

The fact that in each step RADI solves linear systems with $p+m$ right hand side vectors, compared to only *p* vectors in RKSM, may become noticable when *m* is larger than *p*. This effect is shown in Table 3 for CUBE with $m=10$ and $p=1$. Unlike these two methods, EBA can precompute the LU factorization of *A*, and win by a large margin in this test case.

### Example 3

Next, we run the Riccati solvers for the well-known benchmark example CHIP. All coefficient matrices for the Riccati equation are taken as they are found in the Oberwolfach Model Reduction Benchmark Collection [17]. Here we solve the generalized Riccati equation (25).

The cost of precomputing shifts is very high in case of CHIP. One fact not shown in the table is that all algorithms which compute shifts dynamically have already solved the Riccati equation before “RADI—Penzl” has even started.

### Example 4

We use the IFISS 3.2. finite-element package [31] to generate the coefficient matrices for a generalized Riccati equation. We choose the provided example T-CD 2 which represents a finite element discretization of a two-dimensional convection diffusion equation on a square domain. The leading dimension is $n=66049$, with *E* symmetric positive definite, and *A* non-symmetric. The matrix *B* consists of $m=5$ randomly generated columns, and $C=[C_1, \; 0]$ with random $C_1\in {\mathbb {R}}^{5 \times 5}$ ($p=5$).

**Fig. 3:**
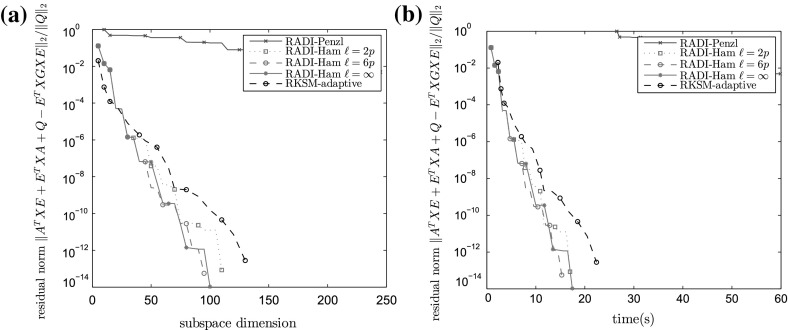
Algorithm performances for benchmark IFISS ($n=66049, m=p=5$). **a** Relative residual versus the subspace dimension used by an algorithm. **b** Relative residual versus time

In this example, the RADI iteration with Penzl shifts converges very slowly. The RADI iteration with dynamically generated shifts are quite fast, and the final subspace dimension is smallest among all methods. On the other hand, the version with residual minimizing shifts does not converge for $\ell =6p, \infty$: it quickly reaches the relative residual of about $10^{-7}$, and then gets stuck by continually using shifts very close to zero. Figure 3 shows the convergence history for some of the used shift strategies.

### Example 5

The example RAIL is a larger version of the steel profile cooling model from the Oberwolfach Model Reduction Benchmark Collection [17]. A finer finite element discretization was used for the heat equation resulting in a generalized CARE $n=317377$, $m=7$, $p=6$, and *E* and *A* symmetric positive and negative definite, respectively. Once again, there is a trade-off between (questionably) better shifts with larger $\ell$ and faster computation with lower $\ell$.

### Example 6

The final example LUNG from the UF Sparse Matrix Collection [12] models temperature and water vapor transport in the human lung. It provides matrices with leading dimension $n=109460$, $E=I$, *A* nonsymmetric, and *B*, *C* are generated as random matrices with $m=p=10$. This example shows the importance of proper shift generation: precomputed shifts are completely useless, while dynamically generated ones show different rates of success. The projection based methods (RKSM, EBA) encountered problems at the numerical solution of the projected ARE. Either the complete algorithm broke down or convergence speed was reduced. Similar issues were encountered at the Galerkin acceleration stage in the Newton-ADI method.

Let us summarize the findings from these and a number of other numerical examples we used to test the algorithms. Clearly, using dynamically generated shifts for the RADI iteration has many benefits compared to the precomputed Penzl shifts. Not only that the number of iterations and running time are reduced, but the convergence is more reliable. Further, there frequently exists a small value of $\ell$ for which one or both of the dynamical shift strategies converge in a number of iterations comparable to runs with $\ell =\infty$, and in far less time. However, an a-priori method of determining a sufficiently small $\ell$ with such properties is still to be found, and a topic of our future research. RADI appears to be quite competitive with other state of the art algorithms for solving large scale CAREs. It frequently generates solutions using the lowest dimensional subspace. Since RADI iterations do not require any orthogonalization nor solving projected CAREs, the algorithm may outperform RKSM and EBA in problems where the final subspace dimension is high. On the other hand, the later methods may have an advantage when the running time is dominated by solving linear systems. It seems that for now, there is no single algorithm of choice that would consistently and reliably run fastest.

## Conclusion

In this paper, we have presented a new low-rank RADI algorithm for computing solutions of large scale Riccati equations. We have shown that this algorithm produces exactly the same iterates as three previously known methods (for which we suggest the common name “Riccati ADI methods”), but it does so in a computationally far more efficient way. As with other Riccati solvers, the performance is heavily dependent on the choice of shift parameters. We have suggested several strategies on how this may be done; some of them show very promising results, making the RADI algorithm competitive with the fastest large scale Riccati solvers.

## Footnotes

1 Note that the generated Penzl shifts come in complex conjugate pairs.

## References

[1] Amodei L, Buchot JM (2010). An invariant subspace method for large-scale algebraic Riccati equation. Appl. Numer. Math..

[2] Benner P, Benner P, Bollhöfer M, Kressner D, Mehl C, Stykel T (2015). Theory and Numerical Solution of Differential and Algebraic Riccati Equations. Numerical Algebra, Matrix Theory, Differential-Algebraic Equations and Control Theory.

[3] Benner P, Bujanović Z (2016). On the solution of large-scale algebraic Riccati equations by using low-dimensional invariant subspaces. Linear Algebra Appl..

[4] Benner P, Kürschner P, Saak J (2013). An improved numerical method for balanced truncation for symmetric second order systems. Math. Comput. Model. Dyn. Syst..

[5] Benner P, Kürschner P, Saak J (2013). Efficient handling of complex shift parameters in the low-rank Cholesky factor ADI method. Numer. Algorithms.

[6] Benner P, Kürschner P, Saak J (2014). Self-generating and efficient shift parameters in ADI methods for large Lyapunov and Sylvester equations. Electr. Trans. Num. Anal..

[7] Benner P, Li JR, Penzl T (2008). Numerical solution of large Lyapunov equations, Riccati equations, and linear-quadratic control problems. Numer. Linear Algebra Appl..

[8] Benner P, Li RC, Truhar N (2009). On the ADI method for Sylvester equations. J. Comput. Appl. Math..

[9] Benner, P., Saak, J.: A Galerkin-Newton-ADI method for solving large-scale algebraic Riccati equations. Preprint SPP1253-090, SPP1253 (2010). http://www.am.uni-erlangen.de/home/spp1253/wiki/index.php/Preprints

[10] Benner P, Saak J (2013). Numerical solution of large and sparse continuous time algebraic matrix Riccati and Lyapunov equations: a state of the art survey. GAMM Mitt..

[11] Bini D, Iannazzo B, Meini B (2012). Numerical Solution of Algebraic Riccati Equations. Fundamentals of Algorithms.

[12] Davis TA, Hu Y (2011). The University of Florida sparse matrix collection. ACM Trans. Math. Softw..

[13] Druskin V, Simoncini V (2011). Adaptive rational Krylov subspaces for large-scale dynamical systems. Syst. Control Lett..

[14] Golub GH, Van Loan CF (2013). Matrix Computations.

[15] Heinkenschloss M, Weichelt HK, Benner P, Saak J (2016). An inexact low-rank Newton-ADI method for large-scale algebraic Riccati equations. Appl. Numer. Math..

[16] Heyouni M, Jbilou K (2009). An extended block Arnoldi algorithm for large-scale solutions of the continuous-time algebraic Riccati equation. Electr. Trans. Num. Anal..

[17] Korvink JG, Rudnyi EB, Benner P, Sorensen DC, Mehrmann V (2005). Oberwolfach benchmark collection. Dimension Reduction of Large-Scale Systems, Lecture Notes in Computational Science and Engineering.

[18] Kürschner, P.: Efficient low-rank solution of large-scale matrix equations. Ph.D. thesis, Otto-von-Guericke-Universität Magdeburg (2016)

[19] Lancaster P, Rodman L (1995). The Algebraic Riccati Equation.

[20] Laub AJ (1979). A Schur method for solving algebraic Riccati equations. IEEE Trans. Autom. Control.

[21] Levenberg N, Reichel L (1993). A generalized ADI iterative method. Numer. Math..

[22] Li JR, White J (2002). Low rank solution of Lyapunov equations. SIAM J. Matrix Anal. Appl..

[23] Lin Y, Simoncini V (2015). A new subspace iteration method for the algebraic Riccati equation. Numer. Linear Algebra Appl..

[24] Massoudi A, Opmeer MR, Reis T (2016). Analysis of an iteration method for the algebraic Riccati equation. SIAM J. Matrix Anal. Appl..

[25] Mehrmann V, Tan E (1988). Defect correction methods for the solution of algebraic Riccati equations. IEEE Trans. Autom. Control.

[26] Overton ML (1992). Large-scale optimization of eigenvalues. SIAM J. Optim..

[27] Penzl, T.: Lyapack Users guide. Technical Report SFB393/00-33, Sonderforschungsbereich 393 Numerische Simulation auf massiv parallelen Rechnern, TU Chemnitz, 09107 Chemnitz, Germany (2000). http://www.tu-chemnitz.de/sfb393/sfb00pr.html

[28] Ruhe A (1994). The rational Krylov algorithm for nonsymmetric eigenvalue problems. III: complex shifts for real matrices. BIT.

[29] Saak, J.: Efficient numerical solution of large scale algebraic matrix equations in PDE control and model order reduction. Ph.D. thesis, TU Chemnitz (2009). http://nbn-resolving.de/urn:nbn:de:bsz:ch1-200901642

[30] Sabino, J.: Solution of large-scale Lyapunov equations via the block modified smith method. Ph.D. Thesis, Rice University, Houston, Texas (2007). http://www.caam.rice.edu/tech_reports/2006/TR06-08.pdf

[31] Silvester, D., Elman, H., Ramage, A.: Incompressible Flow and Iterative Solver Software (IFISS) version 3.2 (2012). http://www.manchester.ac.uk/ifiss

[32] Simoncini V (2007). A new iterative method for solving large-scale Lyapunov matrix equations. SIAM J. Sci. Comput..

[33] Simoncini V (2016). Computational methods for linear matrix equations. SIAM Rev..

[34] Simoncini V, Szyld D, Monsalve M (2014). On two numerical methods for the solution of large-scale algebraic Riccati equations. IMA J. Numer. Anal..

[35] Wachspress E (1988). Iterative solution of the Lyapunov matrix equation. Appl. Math. Lett..

[36] Wachspress E (2013). The ADI Model Problem.

[37] Wong, N., Balakrishnan, V.: Quadratic alternating direction implicit iteration for the fast solution of algebraic Riccati equations. In: Proceedings of International Symposium on Intelligent Signal Processing and Communication Systems, pp. 373–376 (2005)

[38] Wong N, Balakrishnan V (2007). Fast positive-real balanced truncation via quadratic alternating direction implicit iteration. IEEE Trans. Comput.-Aided Des. Integr. Circuits Syst..

